# Graves’ disease with normal or heterogeneous 99mTc uptake: insights into the atypical scintigraphy patterns

**DOI:** 10.20945/2359-4292-2025-0160

**Published:** 2025-10-23

**Authors:** Mainak Banerjee, Hridish Narayan Chakravarti, Debmalya Sanyal, Mukesh Jain, Varnali Chatterjee, Anuska Ghosh, Debasree Biswas

**Affiliations:** 1 Department of Endocrinology, Rabindranath Tagore Hospital, Kolkata, West Bengal, India; 2 RKM Seva Pratisthan & VIMS, Kolkata, West Bengal, India; 3 Department of Endocrinology, Rabindranath Tagore Hospital (RTIICS), Kolkata, West Bengal, India; 4 KPC Medical College & Hospital, Kolkata, West Bengal, India; 5 Division of Nuclear Medicine, Rabindranath Tagore Hospital, Kolkata, West Bengal, India; 6 Department of Nursing, Rabindranath Tagore Hospital, Kolkata, West Bengal, India; 7 Division of Biochemistry, Rabindranath Tagore Hospital, Kolkata, West Bengal, India

**Keywords:** Mild Graves’, TSH receptor antibody, Goitre, 99m-Tc scintigraphy, heterogeneous, distribution

## Abstract

**Objective:**

Graves’ disease (GD), an autoimmune disorder causing hyperthyroidism, is often diagnosed using 99mTc scintigraphy. While increased thyroidal 99mTc uptake with homogenous distribution is typical, atypical patterns (normal or heterogeneous) occur. This study aimed to investigate the determinants of these atypical uptake patterns in GD.

**Subjects and methods:**

Re-trospective records review identified 238 GD patients diagnosed between January 2022 and December 2024. Normal 99mTc uptake (0.4%-3%) and heterogeneous distribution patterns were defined based on scintigraphy. Relevant clinical and biochemical data were compared between typical and atypical pattern groups.

**Results:**

Normal 99mTc uptake was observed in 25/238 (10.5%). Compared to increased uptake, it was associated with lower FT4, T3 levels (p < 0.01) and TRAb levels (p = 0.01) with similar prevalence of heterogeneous distribution (p > 0.05). Compared to homogeneous uptake, heterogeneous uptake subgroup (n = 30/238, 12.6%) had similar TRAb/T3 levels (p > 0.05) with lower FT4 (p = 0.04); and were more likely to have goiter grade > 1 (p < 0.05). Age, gender, smoking and thyroid eye disease were not associated with either atypical uptake pattern. In regression analysis, lower TRAb was associated with normal uptake (OR 0.798, 95% CI 0.660-0.965, p = 0.02), and goitre grade > 1 was associated with heterogeneous uptake (OR 4.34, 95% CI 1.25-15.03, p = 0.01).

**Conclusion:**

Atypical 99mTc uptake patterns were observed in a notable subset of GD. Normal uptake subgroup may reflect a mild evolving disease stage with lower TRAb, while heterogeneous uptake was primarily linked to increased thyroid size. These findings highlight the importance of integrating clinical and biochemical data when interpreting thyroid scintigraphy in suspected GD.

## INTRODUCTION

Graves’ disease (GD), an autoimmune disorder characterized by hyperthyroidism, results from the overproduction of thyroid hormones due to the stimulation of the thyroid gland by autoantibodies (^1^). Technetium-99m pertechnetate (99mTc) scintigraphy is a key diagnostic tool for evaluating thyroid function, offering advantages over 123-Iodine in many countries due to its reduced cost, shorter procedure time, and wider availability (^1^). Contemporary clinical guidelines suggest measuring TSH receptor antibody (TRAb) levels as the initial diagnostic test for hyperthyroidism, followed by thyroid radionuclide scintigraphy if TRAb levels are low or unavailable (^2^,^3^). Nevertheless, these guidelines acknowledge the significant regional variations in clinical practice (^3^). In many settings, clinicians perform scintigraphy, driven by the limited local access to TRAb testing, which can result in increased cost and prolonged turnaround times due to infrequent batch processing (^4^,^5^). Scintigraphy is particularly valuable when radioiodine ablation is planned as definite treatment for GD (^2^,^3^).

A homogenous and increased uptake of Tc-99m in thyroid gland is typical scintigraphic characteristic of GD (^1^). While this finding may obviate the need for TRab testing for diagnostic purposes, it is important to note that the numerical range of “normal” uptake in GD may overlap with that of euthyroid individuals and underlying physiological mechanisms may be distinct (^6^). It remains uncertain whether a normal quantitative 99m-Tc uptake pattern correlates with reduced disease severity regardless of age or gender (^7^,^8^). The occurrence of inhomogeneous distribution of 99m-Tc also warrants careful consideration (^9^,^10^). Heterogeneous uptake might suggest the presence of autonomous nodules, areas of fibrosis, or other structural changes within the thyroid gland, potentially requiring further investigation such as ultrasound with or without fine-needle aspiration biopsy (^9^,^10^). Some studies suggest that demographic parameters might predispose patients to these atypical uptake patterns, but definitive predictors have not been consistently identified (^6^-^9^,^11^). Elucidating the factors contributing to these atypical uptake patterns may provide insights into the disease heterogeneity of GD, which is essential for accurate diagnosis and monitoring of disease progression.

This study aimed to explore the impact of relevant clinical and biochemical variables including age, gender, TRAb levels, and thyroid size, on the normal and inhomogeneous 99m-Tc uptake in GD.

## SUBJECTS AND METHODS

### Patient selection

This was a cross-sectional study conducted at a tertiary care centre. Ethical approval was obtained from institutional ethics committee (NHRTIICSEC/INV/Non-Reg/2025/003).

We reviewed the electronic medical records of consecutive patients who underwent evaluation for hyperthyroidism, defined as suppressed TSH levels with elevated thyroid hormones, between January 2022 and December 2024. Patients were included if they received a diagnosis of Graves’ disease (GD), confirmed by an endocrinologist based on one or more of the following criteria: elevated TRAb levels, and/or typical scintigraphic pattern of increased homogeneous 99mTc uptake. Exclusion criteria were: prior history of anti-thyroid drugs (ATD) or radioiodine ablation, pregnancy, breastfeeding, or refusal to undergo 99mTc uptake scintigraphy.

### Assessment of clinical parameters

The presence of thyroid-associated ophthalmopathy (TED) was determined based on established clinical criteria (^12^). Current smoking status was ascertained through self-reported data documented in prescription records. Retrospective review of patient scintigraphy records was conducted to extract the information on thyroid gland size. The preliminary clinical examination, performed prior to the scintigraphy, included a goiter grading assessment by the attending nuclear medicine physician, which was subsequently cross-checked by another physician. Goiter was graded as “normal/mildly enlarged” (grade 0-1) or “enlarged” (grade > 1). Grade 0 indicated no palpable goiter, grade 1 indicated a goiter visible only with neck extension, and grade > 1 indicated a goiter visible with the neck in a neutral position including those with significant nodularity (^13^).

### Biochemical tests

Blood samples were collected within 48 hours prior to thyroid scintigraphy, before any antithyroid treatment initiation. Laboratory personnel were blinded to patients’ scintigraphy classification. Serum thyroid-stimulating hormone (TSH), free thyroxine (FT4) and triiodothyronine (T3) levels were measured using enhanced chemiluminescence immunoassay (ECLIA) on a Vitros XT 7600 analyzer (Ortho Clinical Diagnostics, Raritan, NJ, USA). Normal reference values were 0.45-4.16 m IU/L, 0.78-2.19 ng/dL, and 1.12-2.08 ng/dL, respectively. TRab was measured using a third-generation electrochemiluminescence immunoassay based on the Roche e411 platform (Roche Diagnostics, Mannheim, Germany) (^14^). The manufacturer kit insert suggested that TRAb titers of > 1.75 IU/L have a sensitivity of 96% and a specificity of 99% in the diagnosis of GD (^14^). The inter-assay coefficients of variation for all analyses were < 5%.

### Scintigraphy and defining atypical 99m-Tc uptake patterns

All patients had a thyroid 99mTc-uptake scintigraphy scan at baseline. The scan was performed using a dual headed gamma camera (GE Discovery 670 DR SPECT-CT, USA) with low-energy, high-resolution collimator and a 20% energy window centered at 140 Kev. Patients were positioned in supine, and the images of thyroid were acquired in anterior and oblique views after 20 minutes of intravenous injection of 4 mCi of Tc-99m pertechnetate according to pre-specified protocol in our centre. Quality control measures, including daily flood field uniformity checks to ensure detector consistency, were performed.

Quantitative 99mTc uptake was calculated as [100 x (thyroid counts - background counts) / injected activity]. The background counts were obtained from a region of interest (ROI) outside the thyroid gland, thyroid counts were obtained from an ROI encompassing the thyroid gland, and the injected activity was determined by measuring the syringe before and after injection (^15^). Quantitative analysis was performed by a nuclear medicine specialist and independently reviewed by a second experienced physician, both blinded to patients’ TRAb status. Inter-observer discrepancies were resolved through consensus. Normal 99mTc uptake was defined as 0.4% to 3%, consistent with the ranges of euthyroid subjects in areas of iodine sufficiency (^16^).

The pattern of radio-labelled tracer distribution was reported as homogenous or heterogeneous, whereas ambiguous cases were resolved after mutual discussion. Heterogeneous uptake was defined as visual evidence of regional variation in radiotracer distribution affecting more than 20% of thyroid parenchyma. Inter-observer agreement for scintigraphy interpretation was substantial (Cohen’s kappa > 0.85). Both interpreters were blinded to biochemical and clinical data.

### Statistical analysis

Normality of continuous variables was assessed using the Shapiro-Wilk test. Continuous data with a normal and non-normal distribution were presented as mean ± SD and median (interquartile range) respectively. Comparison between groups was performed by Mann-Whitney test or student t test as applicable. Categorical data was presented as percentages, and chi-square test was applied to compare the data between two groups. Missing data were handled using listwise deletion. To investigate the independent association of variables with normal 99m-Tc uptake (0.4%-3%) and heterogeneous uptake patterns on scintigraphy in GD, a multivariate binary logistic regression was applied including potential variables based on their biological plausibility and previous literature. We considered potential variables such as age, male gender, body mass index (BMI), current smoking status, thyroid gland size (goitre grade 0-1 *versus* grade > 1) and TRab levels. Multicollinearity was assessed using variance inflation factors (VIFs), and no significant collinearity was detected (VIF < 2 for all variables). The strength of association was assessed using adjusted odds ratios (ORs) with 95% confidence intervals (CIs). The goodness-of-fit of the final model was evaluated using the Hosmer-Lemeshow test. The variance explained by the model was determined using the Nagelkerke R^2^ statistic. A two-sided p-value of < 0.05 was considered significant for all analyses. Statistical analyses was performed using SPSS version 26.

## RESULTS

**Figure 1:**
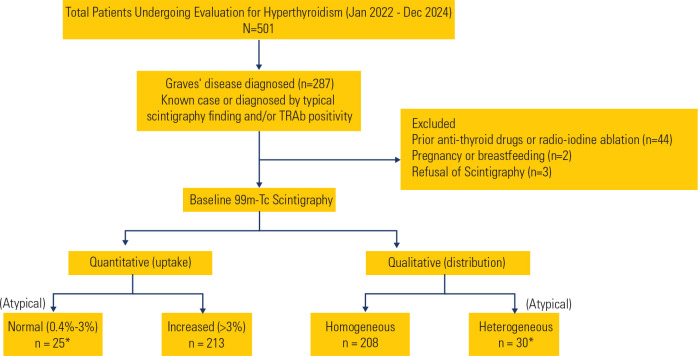
Flowchart of patient selection and classification based on 99mTc-pertechnetate scintigraphy in Graves’ disease.

*Note: 5 patients with normal 99mTc uptake also exhibited a heterogeneous distribution pattern, although this association was not statistically significant (p > 0.05).

Of the 312 patients initially screened for hyperthyroidism during the study period, 74 were excluded due to prior ATD use (n = 28), radioiodine therapy (n = 25), pregnancy (n = 8), breastfeeding (n = 5), and refusal to undergo scintigraphy (n = 8), resulting in 238 patients of GD included in the final analysis (**Figure 1**). Median age of the cohort was 41 years (IQR 31-52); and 64.2% of them were females.

Twenty-five individuals exhibited normal 99mTc uptake in thyroids on scintigraphy, representing 10.5% of the cohort. Compared to the increased uptake group, the normal uptake group did not differ significantly from the increased uptake group in terms of age, gender distribution, body mass index (BMI), presence of thyroid eye disease (TED), thyroid gland size (goiter grade 0-1 *vs.* > 1), or the prevalence of heterogeneous 99mTc distribution (all p > 0.05). Patients with normal uptake had less severe biochemical hyperthyroidism at diagnosis: median FT4 levels 2.6 ng/dL *versus* 3.2 ng/dL (p < 0.001), and median T3 levels 2.7 ng/dL compared to 3.5 ng/dL in those with increased uptake (p = 0.001). Out of those patients for whom a third-generation TRAb assay report was available (n = 83), TRAb levels were significantly lower in patients with normal 99m-Tc uptake GD (n = 20), with a median of 4.8 U/L compared to 7.5 U/L in the increased uptake subgroup (n = 63) (p < 0.05) (**Table 1**).

**Table 1:** Difference in variables in normal 99m-Tc uptake group *versus* the typical increased uptake group in GD

	Increased uptake (n = 213)	Normal uptake (n = 25)	P value
Age (years)	40 [31.5-51]	51 [28-59]	0.255
Females (n, %)	135 (63.4%)	18 (72%)	0.395
Body mass index (kg/m^2^)	24.0 [22.7-25.6]	23.9 [22.2-25.2]	0.979
Current smoking	29 (13.6%)	4 (16%)	0.744
TED	18 (8.5%)	2 (8%)	0.939
FT4 (ng/dL)	3.2 [2.5-4.3]	2.6 [2.2-2.8]	0.007^*^
T3 (ng/dL)	3.5 [2.9-4.1]	2.7 [2.4-3.0]	0.001^*^
TRab (U/L)^	7.5 [4.5-10.4]	4.8 [4.1-6.4]	0.018^*^
Thyroids enlarged Normal or mildly enlarged Moderate-severe	80 (37.6%) 133 (62.4%)	10 (40%) 15 (60%)	0.812
99m-Tc distribution Heterogeneous	25 (11.7%)	5 (20%)	0.239

Categorical data presented as n (%). Continuous data presented as median [IQR].

* Differences significant at p value < 0.05. ^Analysis performed with third generation TRab assays available for 63 and 19 patients in both subgroups respectively.

TED: thyroid eye disease; FT4: free thyroxine; T3: triiodothyronine; TRAb: TSH receptor antibody; GD: Graves’ disease; IQR: interquartile range.

**Figure 2:**
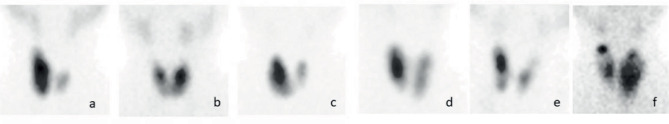
Scintigraphy films of six patients with Graves’ disease showing heterogeneous distribution of 99m-Tc in thyroids.

Note: All patients with scintigraphy images (a-f) were TRAb+ with cold nodule detected on ultrasound in one with a subsequent diagnosis of papillary thyroid carcinoma (2e).

In the same cohort, 208 patients (87.4%) demonstrated homogeneous 99mTc uptake, while 30 patients (12.6%) exhibited heterogeneous uptake (**Figure 2**). There were no significant differences in age, gender distribution, BMI, or TED prevalence between the homogeneous and heterogeneous uptake groups (all p > 0.05). However, patients with heterogeneous uptake were more likely to have enlarged thyroid glands i.e. goiter grade >1 (p < 0.05). Additionally, the heterogeneous uptake group had significantly lower median free T4 (FT4) levels (p < 0.01), but similar T3 levels compared to the homogeneous uptake group (p > 0.05). TRAb levels were similar in patients with heterogeneous 99m-Tc uptake GD (n = 22) *versus* homogenous uptake group (n = 61) **(Table 2**). Of the 30 patients with heterogeneous 99mTc uptake, 25 had thyroid ultrasonography data available. No gross asymmetry in volume between the lobes was observed on USG. Low uptake solid nodularity was identified in 10 patients, and one patient had Marine-Lenhart syndrome (**Figure 2f**). Cysto-solid nodules were found in 2 patients, and pure cysts in another 2 patients. Three individuals were subsequently diagnosed with papillary thyroid carcinoma via fine-needle aspiration biopsy. It is notable that 10 patients (40%) with heterogeneous uptake who underwent USG did not show any focal nodular or cystic lesions, suggesting patchy areas of altered uptake within otherwise morphologically unremarkable parenchyma.

**Table 2:** Difference in variables in heterogeneous 99m-Tc uptake group *versus* the typical homogeneous uptake group in GD

	Homogenous uptake (n = 208)	Heterogeneous uptake (n = 30)	P value
Age (years)	41.5 [31.2-52.0]	44 [28.2-52.2]	0.986
Females (n, %)	134 (64.4%)	19 (63.3%)	0.907
Body mass index (kg/m^2^)	24.0 [22.7-25.6]	23.8 [22.5-24.9]	0.530
Current smoking (n, %)	26 (12.5%)	7 (23.3%)	0.108
TED (n, %)	17 (8.2%)	3 (10%)	0.736
FT4 (ng/dL)	3.4 [2.8-4.4]	2.6 [2.2-3.0]	0.045^*^
T3 (ng/dL)	3.3 [2.8-4.0]	3.2 [2.3-3.8]	0.139
TRab (U/L)^	5.8 [4.1-10.3]	6.6 [4.3-8.5]	0.553
Thyroids enlarged Normal or mildly enlarged Moderate-severe	85 (40.9%) 123 (59.1%)	5 (16.7%) 25 (83.3%)	0.011^*^
Thyroid 99m-Tc uptake Absolute (%)	7.8 [4.7-14.6]	7.3 [4.6-9.2]	0.156

Categorical data presented as n (%). Continuous data presented as median [IQR].

* Differences significant at p value < 0.05. ^Analysis performed with third generation TRab assays available for 61 and 21 patients in both subgroups respectively.

TED: thyroid eye disease; FT4: free thyroxine; T3: triiodothyronine; TRAb: TSH receptor antibody; GD: Graves’ disease; IQR: interquartile range.

Multivariable binary logistic regression, using ‘enter’ method of variable selection, revealed an inverse association between serum TRAb levels and normal 99mTc uptake. Other variables, such as age, gender (males *versus* females), BMI, smoking status (yes *versus* no), TED (yes versus no) and thyroid gland size (goitre grade 0-1 *versus* grade > 1), were not significantly associated with normal 99mTc uptake. Specifically, for each unit increase in TRAb, the adjusted odds of normal 99mTc uptake decreased by 20.2% (adjusted OR 0.798, 95% CI 0.660-0.965, p < 0.05). The Hosmer-Lemeshow goodness-of-fit test indicated a good model fit (χ^2^ = 7.2, df = 8, p = 0.511). The model explained 19.7% of the variance in normal 99mTc uptake (Nagelkerke R^2^ = 0.197).

Likewise, multivariable logistic regression for heterogeneous 99mTc uptake showed that only thyroid gland size was significantly associated. Age, gender, BMI, smoking status, TED, and TRAb levels were not significantly associated with heterogeneous uptake. Patients with enlarged thyroid glands (goiter grade > 1) had a 4.34-fold increased odds of heterogeneous uptake (adjusted OR 4.34, 95% CI 1.25-15.03, p = 0.02). The Hosmer-Lemeshow goodness-of-fit test indicated a good model fit (χ^2^ =13.1, df = 8, p = 0.107). The model explained 14.6% of the variance in heterogeneous 99mTc uptake (Nagelkerke R^2^ = 0.146).

## DISCUSSION

In this study, we explored the characteristics of atypical 99mTc uptake patterns in a cohort of 238 patients with GD. Our findings revealed that 10.5% of the patients exhibited normal 99mTc uptake, while 12.6% demonstrated inhomogeneous uptake. Patients with normal uptake presented with less severe biochemical hyperthyroidism, characterized by lower T4 and T3 levels, along with significantly lower TRAb levels. Conversely, heterogeneous uptake was significantly associated with enlarged thyroid glands (goiter grade > 1).

The prevalence of normal 99mTc uptake in our cohort (10.5%) aligns with existing literature (^6^,^17^,^18^). While quantitative 99mTc uptake measurement may not be routinely performed in all centres, understanding the factors associated with normal uptake patterns could have implications for disease management. It has been previously reported that there can be a negative influence of increasing age or male gender on 99m-Tc uptake (^7^). In fact, age appeared to influence the degree of hyperthyroidism independent of TRAb concentrations in a cohort of GD, indicating a reduction in thyroid responsiveness with ageing (^8^). However, the demographic variables had no association with normal uptake pattern in GD in our cohort. Our observation of lower FT4, T3, and TRAb levels in the normal uptake group suggests that these patients may represent a milder or earlier stage of GD. This finding aligns with the concept that TRAb-mediated thyroid stimulation drives hormone production (^19^). The fact that only a modest proportion (19.7%) of the variance in normal 99mTc uptake was explained by TRAb levels in our regression model highlights the complex interplay of factors influencing thyroid function in GD. While the third-generation TRAb assay used in this study is highly sensitive and specific for GD, it does not distinguish between stimulating, blocking, and neutral TRAbs (^20^). Further research using bioassays that differentiate TRAb subtypes could provide more insight into this relationship.

The presence of heterogeneous 99mTc uptake in 12.6% of our GD patients underscores the importance of careful interpretation of scintigraphy results, which often necessitates ultrasound evaluation (^21^). While homogeneous uptake is a common finding in GD, heterogeneous uptake can suggest presence of underlying structural or functional changes within the thyroid gland as observed in this study. These changes include low-uptake or autonomous nodules, or diffuse parenchymal changes such as micronodular patterns, hyperplasia or fibrosis without clearly defined discrete nodules (^9^,^22^,^23^). This heterogeneity may also reflect areas of varying functional activity in the parenchyma due to autoimmune inflammation (^9^,^22^,^23^).

In heterogeneously functioning thyroids, localized nodular activity may lead to higher conversion (T4 → T3) and synthesis of T3, whereas other inactive parts with aberrant function may cause relatively reduced T4 synthesis. This could explain the discordant biochemical picture in heterogeneous uptake group compared to homogenous uptake group of our study. This contrasts with a previous study that reported lower T3, FT4 and TRAb levels in heterogeneous uptake group compared to homogenous uptake group (^9^).

Our finding of a significant association between heterogeneous uptake and enlarged thyroid glands (goiter grade > 1) supports the concept that areas of varying functional activity may develop within the gland resulting from hyperplasia and hypertrophy of thyroid follicular cells owing to prolonged TRAb stimulation or other factors. However, these structural and functional changes due to long-standing stimulation may not necessarily correlate with TRAb levels at a single point of time, as indicated by similar TRAb levels in heterogeneous uptake group *versus* homogenous uptake group. In a previous study, TRAb levels were usually not considered to be the primary driver of thyroid nodularity or volume (^24^). It is also plausible that the distribution of blood flow within a larger gland might be uneven, leading to a differential uptake of 99mTc in various regions.

The present study has several limitations. The cross-sectional design restricts our ability to establish causal relationships. Consistent with real-world clinical practice, USG data was not available for patients where scintigraphy and/or TRAb results clearly indicated GD. Consequently, a comparative analysis correlating detailed morphological changes in USG across all uptake patterns could not be performed. Therefore, the comparative analysis with regard to thyroid size between two groups relied solely on the clinical goiter grading. However, it is important to note that for cases demonstrating heterogeneous 99mTc uptake patterns, where USG was performed, a significant proportion of patients did present with nodularity, and no instances of significant asymmetry in thyroid lobe morphology were observed. Third-generation TRAb assays were not available for all patients, particularly those with typical scintigraphic characteristics for whom TRAb testing was not deemed necessary for diagnostic purposes. Furthermore, TRAb test results from other external laboratories were excluded from the analysis to minimize potential bias arising from inter-laboratory variability in assay methodologies. Thus, the relatively small size of these subgroups with available TRAb (20 in normal uptake group and 22 in heterogeneous uptake group) might have underpowered the analysis, limiting our ability to detect any subtle influence of TRAb on heterogeneous 99mTc distribution, independent of thyroid size (goiter grade > 1). Nonetheless, lower TRAb levels consistent with less severe biochemical hyperthyroidism were observed in patients with normal 99mTc uptake. It may suggest that this subset of patients could be managed with lower doses of anti-thyroid medications or may be candidates for a trial of early withdrawal of therapy. Conversely, patients with heterogeneous uptake and goiter may require closer monitoring for structural changes and potential interventions like radioiodine therapy or surgery. Hence, longitudinal multi-centre studies with larger cohorts are needed to evaluate the long-term outcomes and response to treatment in GD patients with atypical uptake patterns. Future studies may also investigate the role of ultrasound elastography in characterizing thyroid nodules in patients with heterogeneous uptake.

In conclusion, atypical 99mTc uptake patterns, including normal (10.5%) and heterogeneous (12.6%) distributions, are observed in a subset of patients with GD. Factors such as age, gender, and smoking status did not significantly influence these patterns. Our findings indicate that normal 99mTc uptake is associated with milder disease and lower TRAb levels, while thyroid size is a critical determinant of heterogeneous uptake. These insights may contribute to a better understanding of pathophysiology behind disease heterogeneity, enhance clinical decision-making and improve diagnostic accuracy in managing patients with Graves’ disease.

## Data Availability

datasets related to this article will be available upon request to the corresponding author.
